# Sex-specific enhancement of palatability-driven feeding in adolescent rats

**DOI:** 10.1371/journal.pone.0180907

**Published:** 2017-07-14

**Authors:** Andrew T. Marshall, Angela T. Liu, Niall P. Murphy, Nigel T. Maidment, Sean B. Ostlund

**Affiliations:** 1 Department of Anesthesiology and Perioperative Care, Center for Addiction Neuroscience, University of California, Irvine, Irvine, California, United States of America; 2 Hatos Center, Department of Psychiatry and Biobehavioral Sciences, Semel Institute for Neuroscience and Human Behavior, University of California, Los Angeles, Los Angeles, California, United States of America; Queens College, UNITED STATES

## Abstract

It has been hypothesized that brain development during adolescence perturbs reward processing in a way that may ultimately contribute to the risky decision making associated with this stage of life, particularly in young males. To investigate potential reward dysfunction during adolescence, Experiment 1 examined palatable fluid intake in rats as a function of age and sex. During a series of twice-weekly test sessions, non-food-deprived rats were given the opportunity to voluntarily consume a highly palatable sweetened condensed milk (SCM) solution. We found that adolescent male, but not female, rats exhibited a pronounced, transient increase in SCM intake (normalized by body weight) that was centered around puberty. Additionally, adult females consumed more SCM than adult males and adolescent females. Using a well-established analytical framework to parse the influences of reward palatability and satiety on the temporal structure of feeding behavior, we found that palatability-driven intake at the outset of the meal was significantly elevated in adolescent males, relative to the other groups. Furthermore, although we found that there were some group differences in the onset of satiety, they were unlikely to contribute to differences in intake. Experiment 2 confirmed that adolescent male rats exhibit elevated palatable fluid consumption, relative to adult males, even when a non-caloric saccharin solution was used as the taste stimulus, demonstrating that these results were unlikely to be related to age-related differences in metabolic need. These findings suggest that elevated palatable food intake during adolescence is sex specific and driven by a fundamental change in reward processing. As adolescent risk taking has been hypothesized as a potential result of hypersensitivity to and overvaluation of appetitive stimuli, individual differences in reward palatability may factor into individual differences in adolescent risky decision making.

## Introduction

Adolescence is associated with a heightened propensity for substance abuse and other risky behaviors [1–4], a phenomenon that seems to be particularly apparent in males [5]. It is widely believed that this penchant for risk taking is the product of an ontogenetic change in reward processing [3, 6]. While there is some evidence that reward processing may actually be attenuated during adolescence (e.g., [3, 7]), the majority of recent studies support what is essentially the opposite view: that adolescents engage in risky behavior because they *overvalue* rewarding stimuli (e.g., [6]). For example, not only do adolescent rats exhibit suboptimal decision making [8] and greater willingness to exert effort for palatable food rewards relative to adults [9, 10], they also exhibit elevated consumption of palatable food [10] and increased hedonic taste reactivity to palatable food stimuli [11].

Although these latter findings suggest that the emotional component of feeding is altered during adolescence, the nature and scope of such changes remain poorly understood. For instance, animal research on this topic has focused almost exclusively on males, even though there have been numerous reports of sex differences in risk-taking behavior (for a review, see [12]). Findings that adult male and female rats differ in their consumption and preference for palatable stimuli [13] suggest that sex may be an important factor in the impact of adolescence on reward processing and its influence over feeding behavior.

The current study evaluated changes in reward processing during adolescence for both sexes by assessing consumption of a palatable sweetened condensed milk solution (SCM). Palatable reward processing may serve as a good initial metric for evaluating how appetitive stimulus encoding impacts risk-taking in adolescence. Our basic approach was adapted from a recent quasi-longitudinal study [10], which found a pronounced, but transient, increase in SCM intake (controlling for body weight) during male puberty. In addition to characterizing SCM intake in both male and female rats during adolescence and adulthood, we also applied a sophisticated analytical approach to focus more directly on palatability-driven food consumption. Although a variety of psychological and physiological processes contribute to the control of feeding behavior, a longstanding distinction has been made between the facilitatory influence of palatability (i.e., gustatory/orosensory reward) that promotes initial feeding and the negative gastrointestinal feedback (i.e., satiety) that limits further intake following periods of extended feeding [14–16]. It is therefore important to consider the possibility that differences in total palatable food intake observed across ages may be related to differences in satiety, rather than differences in reward evaluation. A straightforward approach for parsing the influence of palatability and satiety on feeding was described by Davis and Levine (14). Specifically, it was shown that palatable fluid food intake is well characterized as a decaying exponential function [α*exp(-(β*Time))] (see [14]). Here, α corresponds to the initial rate of licking (i.e., the function’s y-intercept), and was shown to be directly related to the palatability (e.g., sweetness or lack of bitterness) of the fluid. In contrast, β refers to the rate of change in lick rate over time (i.e., the slope), and was found to be a useful measure of the suppressive influence of satiety on feeding.

Experiment 1 applied this analytical approach to SCM milk consumption in free feeding (non-deprived) rats. It is likely that SCM intake under these conditions is primarily driven by orosensory reward, as opposed to its nutritional value. However, given the heightened energetic demands associated with adolescent development, Experiment 2 was conducted to assess whether similar effects would be observed with a calorie-free saccharin solution.

## Experiment 1

### Materials and methods

#### Animals and apparatus

Forty-three experimentally naïve Long Evans rats were used in this experiment: 13 adolescent males, 11 adolescent females, 9 adult males, and 10 adult females. Rats were derived from a local colony and weaned at PND 19–20. They were group-housed (2–3 rats per cage) and had *ad libitum* access to both food and water throughout the experiment. The colony room was maintained on a standard 12:12 hr light:dark schedule (lights on at approximately 6:30 am). All experimentation occurred during the light phase. Husbandry, experimental, and euthanasia procedures were approved by the UC Irvine Institutional Animal Care and Use Committee (IACUC) and were in accordance with the National Research Council Guide for the Care and Use of Laboratory Animals. Animals in this experiment were euthanized via CO2 exposure.

Behavioral testing occurred in standard operant chambers (Med Associates; St. Albans, VT, USA) housed within sound- and light-attenuating boxes. Each chamber was equipped with a stainless-steel grid floor, two stainless steel walls (front and rear), and a transparent polycarbonate side wall, ceiling, and door. For this experiment, a drinking bottle containing 10% SCM was attached outside of each chamber, so that a stainless-steel sipper tube could be carefully positioned near a hole in the rear chamber wall. A concentration of 10% SCM was chosen to maximize palatability while minimizing analytical complications due to SCM viscosity (i.e., less accurate licking metrics due to SCM being too viscous). A contact lickometer (ENV-250B; Med Associates) was attached to the sipper tubes to record individual licking events. A houselight mounted at the top of the rear chamber wall provided continuous illumination.

#### Procedure

**Fig 1:**
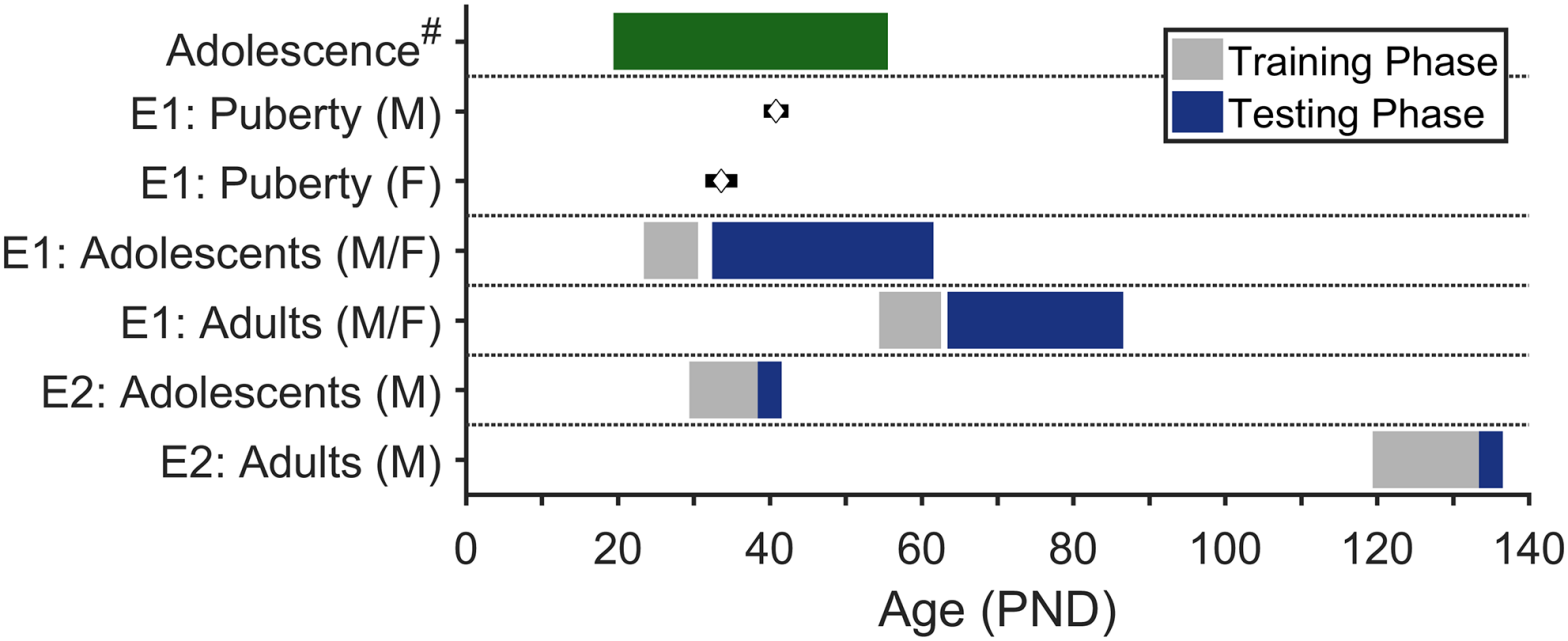
Experimental timeline. The top row corresponds to previously proposed age ranges of adolescence in male and female rats. The second and third rows show the mean (± 1 SD) of puberty onset for the adolescent rats in Experiment 1. The bottom four rows correspond to the ages at which training and testing occurred in Experiments 1 and 2 for adolescent and adult rats. # = Spear (3).

During the experiment, adolescent rats were examined daily for puberty onset, which was indicated by preputial separation (males) and vaginal opening (females) [17]. Mean (SEM) puberty onset (PND) was 40.85 (0.45) for males and 33.64 (0.70) for females. Fig 1 presents an overview of the timelines for Experiments 1 and 2 and how they relate to these estimates of puberty onset and previously reported estimates of the full adolescent window for rats, which is also presumed to begin earlier in females [3].

All rats were initially handled for 3 days prior to the onset of behavioral procedures. Rats were then pre-exposed to SCM for 4 consecutive days to familiarize them with both the SCM and the testing procedure. The first day of pre-training consisted of a single 2-h period of access to SCM in the home cage. On each of the next two days, rats received a 30-min session in which SCM was continuously available in the test chamber, followed by an additional 2-h period of access in their home cage. The final day of pre-training involved only 30-min of SCM access in the test chamber. Pre-training was followed by a series of 30-min consumption sessions conducted in the test chambers to measure changes in SCM intake over time, with 3–4 day intervals between tests (e.g., the interval between the first and third test session was one week). Adolescent and adult rats were given a total of 9 tests (PND 32–61) and 7 tests (PND 64–86), respectively. While this repeated testing ensured that the data were randomly sampled across the females’ estrous phases, a recent meta-analysis suggested that asynchronous estrous phases in female rodents does not elicit any more variability in behavior (including feeding behavior) than that of male rats [18, 19], alleviating the need to consistently monitor estrous stages.

#### Data analysis

All summary measures were obtained from the raw data using MATLAB (The MathWorks; Natick, MA, USA), and analyzed with mixed-effects regression models [20], the use of which represents a transition to a new recommended analytical framework in psychology and neuroscience research [21]. Mixed-effects models are comparable to repeated-measures regression analyses, and allow for parameter estimation per manipulation condition (fixed effects) and the individual (random effects) [20, 22–25]. In contrast to ANOVAs, mixed-effects regression models (1) effectively handle missing data and (2) permit the inclusion of categorical and continuous predictors in the same analysis [26], thus allowing detection of group-level changes across ordered data samples (i.e., continuous time points) while also accounting for corresponding individual differences. Model selection was performed using the Akaike information criterion (AIC), in which the doubled negative log likelihood of the model is penalized by twice the number of estimated parameters (see [27]). Categorical predictors were effects-coded (i.e., codes sum to 0), and continuous predictors were mean-centered [28]. Post hoc analyses were performed using the *coefTest* function (i.e., *F*-tests) in MATLAB; mixed-effects models are inherently conservative and circumvent the need for multiple comparisons corrections [29]. Further details of the analyses are provided in the corresponding results sections.

Total SCM consumption, normalized for body weight (g/kg), was determined by subtracting the weight of the bottle at the end of the session from its weight at the beginning of the session, correcting for approximate spillage. Artifacts in the licking data due to occasional short-circuiting of the lickometer system were identified (i.e., inter-lick intervals less than or equal to 50 ms) and excluded from the analysis. Entire sessions were excluded if 20% or more of recorded licks were identified as artifacts, which rarely occurred (~1.5% of sessions). Given that lick volumes for rats average between 4–8 μl and are unlikely to exceed 10 μl [30, 31], we excluded data from sessions in which estimated average lick volume was greater than this maximum cutoff value (which also occurred rarely; ~8.3% of sessions). We considered that such high apparent volumes reflected inaccuracies due to spillage and/or excessive exclusion of apparent artifacts in the licking data. Aside from the test sessions that were excluded based on these criteria, all test sessions (9 for adolescents, 7 for adults) were included in the analysis.

### Results and discussion

#### Testing: Overall consumption

**Fig 2:**
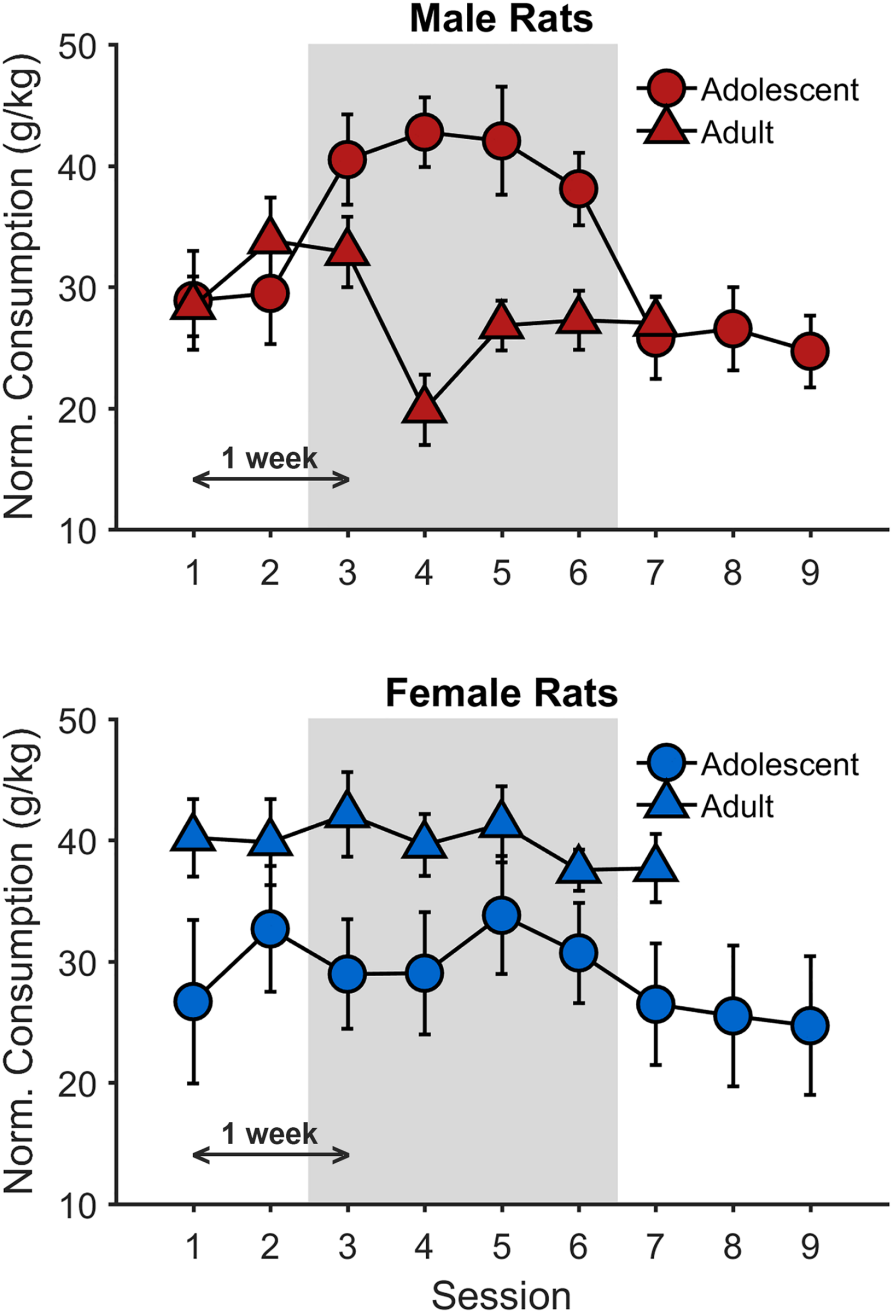
Normalized SCM consumption in Experiment 1. The data are group means +/- 1 between-subjects SEM. The abscissa is session, and the ordinate is SCM consumption per kilogram body weight (i.e., normalized SCM consumption). The shaded area of each panel corresponds to the test sessions in which adolescent males showed elevated normalized SCM consumption relative to earlier and later sessions. The purpose of the shading in the bottom panel is to facilitate comparison. There was a 3–4 d interval between consecutive sessions (i.e., a one-week interval between odd-numbered test sessions and between even-numbered test sessions). Subsequent analyses of all rats’ data in Experiment 1 (see *Testing*: *Within-session Consumption Rate*) only included the data in the sessions within the shaded panels (i.e., Sessions 3–6). See Table 1 for the corresponding statistical analyses results.

Fig 2 shows mean SCM consumption (± 1 SEM), normalized to body weight, as a function of test session and group. Normalized consumption was similar in adolescent and adult male rats early and late in training (i.e., Sessions 1, 2, and 7). However, adolescent males exhibited a substantial yet transient increase in normalized SCM intake during Sessions 3 through 6, which was not apparent in adult males or in either group of female rats. These latter groups exhibited relatively consistent intake levels across sessions (Fig 2). This period of elevated SCM intake, highlighted by a shaded gray region in the male rats’ figure panel (and in the female rats’ figure panel for ease of comparison), corresponds roughly to early- to mid-puberty for male rats, corroborating previous research showing a significant increase in normalized SCM intake in adolescent male rats during a similar developmental window [10]. More specifically, for the adolescent males, Session 3 corresponds to PND 40, and, given the 3–4 d intervals between tests, Session 6 corresponds to PND 51; thus, Sessions 3–6 (PND 40–51) refer to the ages of early to mid-puberty in adolescent male rats [3, 10, 32]. The apparent decrease in consumption for adult male rats following the third test (Session 4) was largely driven by minimal SCM intake by a single animal in that session. It is also notable that adult females exhibited greater normalized SCM intake across sessions compared to adolescent females and adult males (Fig 2). As normalized data may mask whether the effects are due to atypical changes in the numerator (i.e., consumption) or denominator (i.e., body weight), we also evaluated whether changes in body weight or raw consumption could reasonably account for the results. However, the substantial increase in normalized SCM consumption in adult females versus adult males was not driven by differences in raw SCM consumption (S1 Fig, top); adult males and females consumed similar SCM amounts but females consumed more relative to their body weights, which were considerably lower than those of adult males (S1 Fig, bottom). Furthermore, this increase specific to adolescent males was not governed by changes in global food intake per kilogram body weight, as this pattern was not observed in adolescent males’ normalized chow and water consumption measured weekly throughout testing (S2 Fig). Accordingly, these data suggest that the elevation in normalized SCM intake for adolescent males is specific to *palatable* reward consumption.

Previous research has shown a non-monotonic relationship between SCM intake and session (i.e., age) in adolescent rats [10], so the current analysis included session^2^ as a factor to determine if there was a significant quadratic component to the data as a function of session. Linear mixed-effects models involved 337 observations. The best-fitting fixed-effects structure included the overall intercept as well as the full factorial model of Age Group × Sex × Session^2^. The random-effects structure included by-subject intercepts and by-subject slopes of session and session^2^. In-text reporting of results focused on the theoretically important results, but full model output is shown in Table 1.

**Table 1:** Linear mixed-effects model output for the analysis of normalized SCM consumption in Experiment 1.

	*t*(325)	*P*	*b*	95% CI
Intercept	24.64	< .001	34.29	[31.55, 37.03]
Sex (M/F)	0.77	.440	1.08	[-1.66, 3.81]
Age Group (Adult/Adolescent)	0.56	.574	0.78	[-1.95, 3.52]
Session	-1.24	.214	-0.47	[-1.23, 0.28]
Sex * Age Group	-4.11	< .001	-5.72	[-8.46, -2.99]
Sex * Session	-0.17	.867	-0.06	[-0.81, 0.69]
Age Group * Session	0.47	.641	0.18	[-0.57, 0.93]
Session^2^	-2.46	.015	-0.30	[-0.54, -0.06]
Sex * Age Group * Session	0.06	.950	0.02	[-0.73, 0.77]
Sex * Session^2^	0.44	.661	0.05	[-0.19, 0.30]
Age Group * Session^2^	-2.42	.016	-0.30	[-0.54, -0.06]
Sex * Age Group * Session^2^	2.28	.023	0.28	[0.04, 0.52]

Note: continuous variables were mean-centered, and categorical variables were effect coded with Males/Females (Sex) as -1/+1 and Adult/Adolescent (Age Group) as -1/+1.

The goal of this analysis was to determine the effects of sex and age group on normalized SCM consumption as a function of session. There was no main effect of sex, *t*(325) = 0.77, *p* = .440, or age group, *t*(325) = 0.56, *p* = .574, but there was a significant Age Group × Sex interaction, *t*(325) = -4.11, *p* < .001; post hoc analyses indicated that adolescent males exhibited greater SCM intake than adult males, *p* = .001, while adolescent females exhibited reduced SCM intake compared to adult females, *p* = .013. Analysis also revealed a main effect of session^2^, *t*(325) = -2.46, *p* = .015, and a significant Age Group × Sex × Session^2^ interaction, *t*(325) = 2.28, *p* = .023, indicating that the significant quadratic component of session^2^ was moderated by age group and sex. Post hoc pairwise comparisons of the Age Group × Sex × Session^2^ interaction revealed that adolescent males exhibited significantly greater negative quadrature compared to all other groups, *p*s ≤ .038, while the other groups did not significantly differ from one another, *p*s ≥ .174. Furthermore, the main effect of session and the interactions between session and sex and/or age group were not significant (*p*s ≥ .214), indicating that there were no significant global linear trends. Therefore, these data reveal a unique interaction between age and palatable food intake in adolescent males. While this age-related result is seemingly unique to adolescent males, previous research has identified earlier puberty onset in adolescent females [3, 32]. Indeed, early to mid-puberty in adolescent female rats would have overlapped with these rats’ first test sessions. Accordingly, a large initial spike followed by a subsequent decrease in normalized palatable reward consumption in adolescent females would have mirrored the data patterns observed in adolescent males, but this was not observed (Fig 2). Future investigation is warranted to determine whether adolescent females exhibit such increases in normalized palatable reward consumption during early to mid-puberty given similar training histories as the adolescent males in this experiment. That adolescent females do not show such an exaggeration in normalized SCM consumption at identical ages does provide insight into the moderation of age and pubertal stage on potential sex differences in palatable reward consumption.

A second set of post hoc analyses was conducted to determine the sessions corresponding to the increase in adolescent males’ normalized SCM intake. In this case, a linear mixed-effects model included intercept and session as fixed effects and a by-subject intercept as a random effect. To address the goals of this specific test of adolescent male consummatory behavior, session was treated as a categorical predictor. This analysis revealed (1) a significant increase in normalized SCM consumption from Session 2 to Session 3 (PND 36 to 40), *p* = .001, (2) a significant decrease in normalized SCM consumption from Session 6 to Session 7 (PND 51 to 54), *p* < .001, and (3) that the transient increase in normalized SCM consumption was relatively constant throughout Sessions 3–6 (PND 40–51), *p* = .206. Therefore, the adolescent males’ palatable reward intake relative to their body weights increased to an elevated baseline level during the period of development corresponding to early to mid-puberty (i.e., PND ~40–50). Indeed, while changes in adults’ and adolescent females’ raw SCM consumption were relatively proportional to those in body weight (S1 Fig), these data indicate that the adolescent males’ raw SCM consumption in the intermediate sessions significantly exceeded what would have been predicted by changes in body weight as seen in the other groups.

#### Testing: Within-session consumption rate

The tendency for adolescent male rats to show a transient elevation in normalized SCM consumption (Fig 2) corroborates a previous finding in male rats [10] and, when taken together, provides evidence of potential sex specificity of this effect (see General Discussion). Thus, male adolescence (specifically during the timeframe corresponding to early to mid-puberty) may be a unique developmental period of discordant body-weight versus palatable reward-intake patterns. However, an analysis of total palatable reward intake does not itself provide a sufficiently precise measure of the influence of orosensory reward on feeding [16]. As described above, the rate of consumption within an access period decays with access time [33]; initial rates of consumption reflect the subjective palatability of the reward (i.e., hedonic value [34]) and the decay rate represents negative feedback related to the induction of satiety [14, 15]. Individual differences in consumption can be explained by changes in either or both processes [16]. Accordingly, we conducted additional analyses of within-session changes in feeding during Sessions 3–6 (shaded area, Fig 2; PND 40–51 for adolescents) to determine whether this elevation of SCM intake during male adolescence was related to a change in reward or satiety processing.

**Fig 3:**
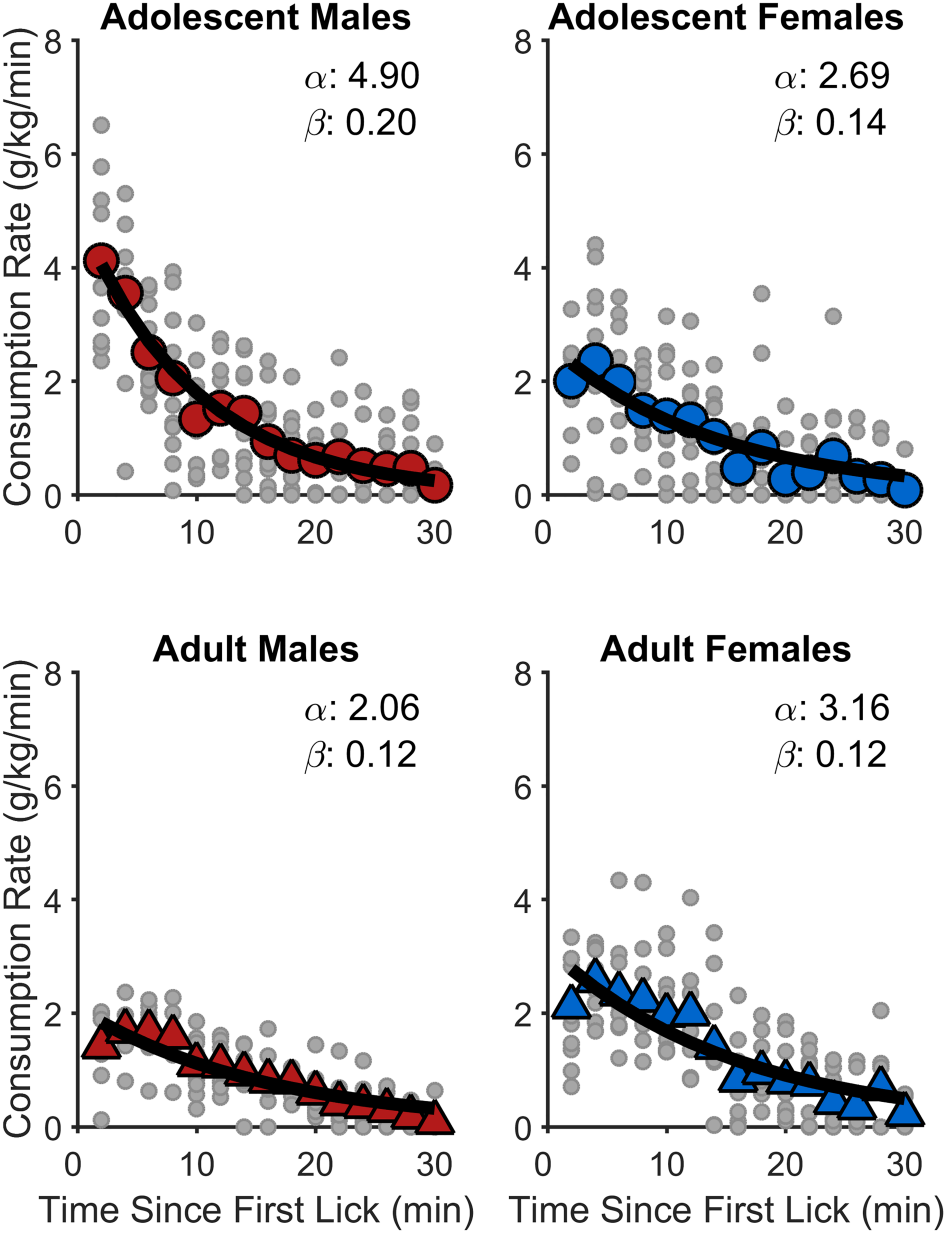
Normalized within-session SCM consumption rates in Experiment 1. The large data points reflect group mean consumption rates in 2-min bins for adolescent males (top-left), adolescent females (top-right), adult males (bottom-left), and adult females (bottom-right). The small shaded data points within each panel represent data from individual rats within that group. The thick line is the best-fitting negative exponential function. The within-panel α and β values are the fitted values of the corresponding exponential function (i.e., α is the y-intercept and a metric for reward palatability; β is the function’s decay and a metric for the induction of satiety). Adolescent males exhibited significantly greater initial consumption rates (α) and decay rates (β) than each of the other three groups, *p*s < .003.

Fig 3 shows the normalized consumption rates as a function of session time relative to the rats’ first lick. In this experiment, within-session consumption rate was determined by multiplying licking rate (i.e., licks per minute) by the average lick volume (ml/lick) for that session, which was then divided by the rat’s body weight (kg) (see [35]). Lick volumes were determined by dividing the total amount of fluid consumed within a session by the total number of licks performed in that session. While lick volumes may undergo modest changes over time during free feeding sessions, it is conventional to assume that lick volumes are relatively constant ([36–41]but see [41]). Consumption rate was computed and plotted in 2-min bins. As seen in Fig 3, all age groups showed evidence of a satiety-like decline in SCM intake over time during test sessions. More importantly, however, it appears that initial consumption rates (i.e., y-intercept) were substantially elevated in adolescent males, relative to other groups.

Nonlinear mixed-effects models fitted to these data included 2,160 observations. As described above, the goal of this analysis was to parse out the positive and negative influences of reward palatability and satiety, respectively, on rats’ palatable reward consumption. Accordingly, the equation used to fit the data was: Consumption Rate ~ α*exp(-(β*Time)), in which α was the initial consumption rate (palatability) and β was the consumption decay rate (satiety) [14]. Nonlinear mixed-effect model fitting was performed in R [42], and significant interactions were further tested with simultaneous *t*-tests in R’s *multcomp* package [43, 44]. The fixed-effects structure for both α and β included the overall intercept, age group, sex, and Age Group × Sex. For both α and β, the random-effects structure only included by-subjects intercept. Full model output is shown in Table 2.

**Table 2:** Nonlinear mixed-effects model output for the analysis of normalized SCM consumption rates in Experiment 1 and normalized saccharin consumption rates in training of Experiment 2.

	*t*(df)	*P*	*b* (*SE*)
Experiment 1 [SCM] (df = 2110)			
α (Initial Consumption Rate)			
Intercept	20.64	< .001	3.20 (0.15)
Age Group (Adult/Adolescent)	3.83	< .001	0.59 (0.15)
Sex (M/F)	-1.80	.073	-0.28 (0.15)
Age Group * Sex	-5.34	< .001	-0.83 (0.15)
β (Consumption Decay Rate)			
Intercept	17.52	< .001	0.15 (0.01)
Age Group (Adult/Adolescent)	2.74	.006	0.02 (0.01)
Sex (M/F)	-1.94	.053	-0.02 (0.01)
Age Group * Sex	-2.00	.046	-0.02 (0.01)
Experiment 2 [Saccharin] (df = 305)			
α (Initial Consumption Rate)			
Intercept	13.68	< .001	2.27 (0.17)
Age Group (Adult/Adolescent)	7.91	< .001	1.31 (0.17)
β (Consumption Decay Rate)			
Intercept	10.91	< .001	0.08 (0.01)
Age Group (Adult/Adolescent)	7.75	< .001	0.06 (0.01)

Note: categorical variables were effect coded with Males/Females (Sex) as -1/+1 and Adult/Adolescent (Age Group) as -1/+1.

Analysis revealed a significant Age Group × Sex interaction on initial consumption rates (α), *t*(2110) = -5.34, *p* < .001. Post hoc tests revealed that adolescent males exhibited significantly greater initial consumption rates than each of the other three groups, *p*s < .001. Adolescent females did not differ from adult males or adult females, *p*s ≥ .153, but adult females did exhibit significantly greater initial consumption rates than adult males, *p* = .013. There was also a significant Age Group × Sex interaction on consumption rate decay (β), *t*(2110) = -2.00, *p* = .046. Adolescent males exhibited significantly greater decays in consumption rates than all other groups, *p*s ≤ .003, but there were no significant differences in consumption rate decay between these other groups, *p*s ≥ .581. Therefore, these analyses indicate that adolescent males differed from the other groups in terms of both their initial consumption rate (palatability) and their rate of satiety-related decay in feeding over time. It is worth noting that the significantly higher decay rates in adolescent males actually correspond to a *stronger*–not weaker—influence of satiety, and is therefore unlikely to explain their elevated SCM intake (Fig 2). Nevertheless, further analyses were conducted to determine whether individual differences in SCM intake were best predicted by individual differences in orosensory reward processing (α) or satiety (β).

**Fig 4:**
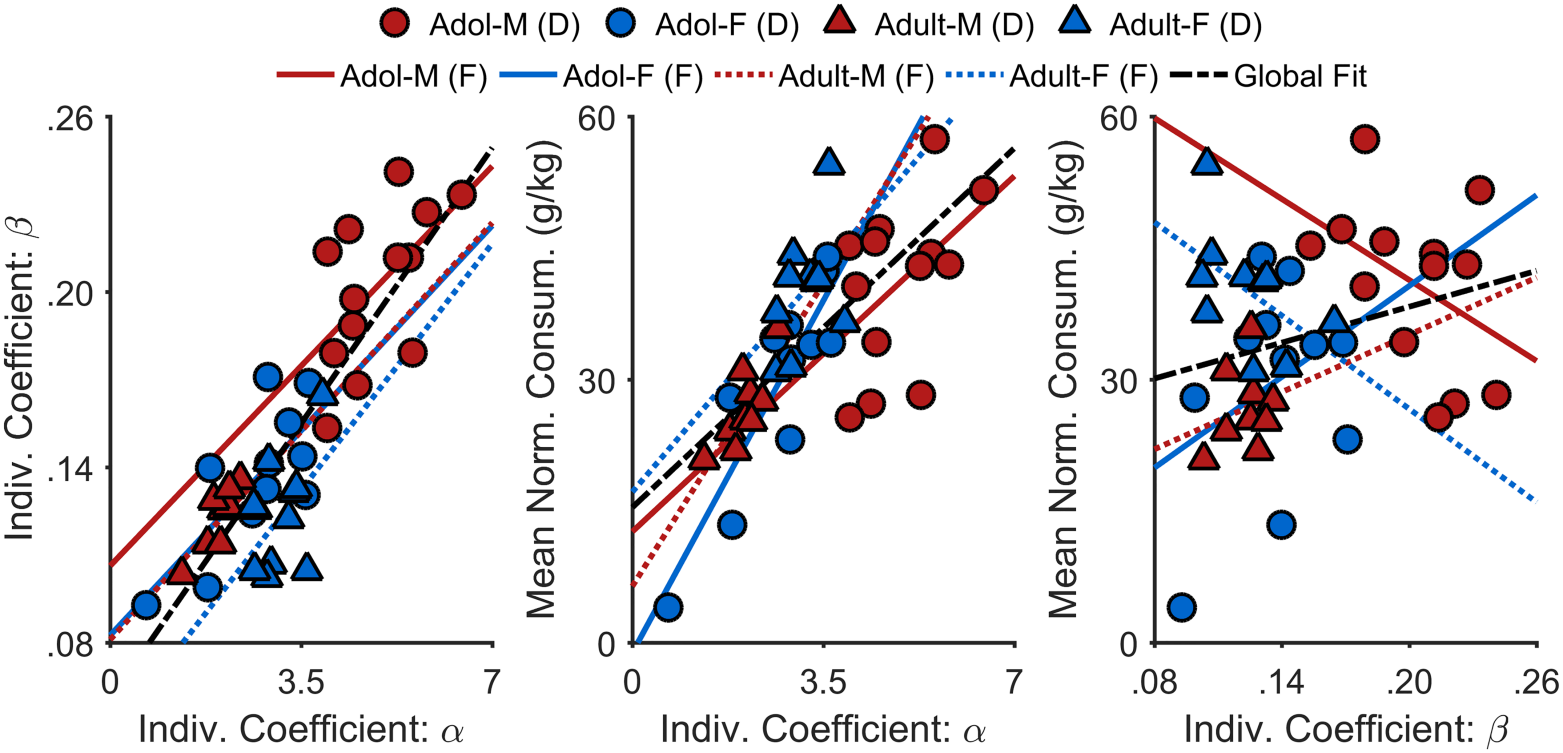
Bivariate scatter plots of individual rats’ mean normalized SCM consumption, initial consumption rates (α), and consumption rate decay (β) in Experiment 1. Each data point represents an individual rat, identified with respect to its sex and age group. The solid and dotted lines are the best-fitting regression lines for each groups’ data. The thick dashed line represents the best-fitting regression line of the data collapsed across groups. There were significant positive relationships between α and β, *p* = .001 (left), and between α and mean normalized SCM intake (middle), *p* < .001. Note: The linear fits are extended beyond the range of the data points for ease of visual interpretation. Adol-M = adolescent-males; Adol-F = adolescent-females; Adult-M = adult-males; Adult-F = adult-females; (D) = data; (F) = linear fit.

Individual α and β values were computed using the fixed and random effects values from the non-linear mixed-effects model and an individual consumption index was operationally defined as the mean normalized SCM intake over Sessions 3–6 (shaded area, Fig 2; i.e., early to mid-puberty in adolescent males). Fig 4 shows bivariate scatter plots of individual α values, individual β values, and mean normalized SCM intake. To determine whether normalized SCM consumption was better predicted by individual differences in orosensory reward processing (α) or satiety signaling (β), three multiple regression analyses were performed, each including the full factorial of Age Group × Sex × Individual α (or β) Coefficients; all main effects and interactions were entered simultaneously into the model. Fig 4 (left) shows the relationship between individual α and β coefficients. As in previous research (see [15, 45]), there was a significant positive relationship between α and β, *b* = 0.02, *t*(35) = 3.50, *p* = .001. The positive relationship was maintained across groups, as indicated by the lack of significant moderation of this relationship by age group and/or sex, |*t*s(35)| ≤ 0.20, *p*s ≥ .839. This finding reflects a basic interdependence between palatability and satiety when assessed under free feeding conditions, in that the rate of satiety depends on the rate of initial feeding [46]. It also suggests that elevated rate of decay in feeding in the adolescent males may be secondary to their heightened rate of initial feeding.

Analysis also revealed a significant positive relationship between α and mean normalized SCM intake (Fig 4, middle), *b* = 8.60, *t*(35) = 3.89, *p* < .001, which was also not moderated by the rats’ age group and/or sex, *t*s(35) ≤ 0.94, *p*s ≥ .356. Finally, there was neither a systematic relationship between β and mean normalized SCM intake (Fig 4, right), *b* = -12.44, *t*(35) = -0.14, *p* = .890, nor moderation of such a relationship by age group and/or sex, *t*s(35) ≤ 1.71, *p*s ≥ .096. Therefore, the results in Fig 4 suggest that, under our testing conditions, individual differences in SCM intake were best characterized by individual differences in initial consumption rates (α) rather than satiety-related decay in feeding (β), consistent with previous research [47]. Altogether, these results collectively suggest that the heightened normalized SCM intake in adolescent males was driven by an increased sensitivity to the orosensory properties of SCM reward rather than a deficit in satiety.

## Experiment 2

The results of Experiment 1 indicate that adolescent males, but not adolescent females, exhibit a transient elevation in palatable food intake and demonstrate that this effect is likely related to a heightened sensitivity to orosensory reward and not a resistance to satiety. While the sex specificity of this result may be influenced by sex differences in age relative to puberty at testing, the results of Experiment 1 do illuminate potential mechanisms for maladaptive reward-seeking behavior in adolescents (i.e., greater hedonic value attributed to reward) (see [3]).

One noteworthy aspect of the palatable SCM solution used in Experiment 1 and in similar studies in the literature [10] is its high caloric value. It is therefore possible that the elevated SCM intake observed during male adolescence is at least partially related to the heightened calorie requirements associated with this developmental period [48]. Experiment 2 was therefore conducted to investigate whether adolescent male rats would show similar elevation in reward intake when consuming saccharin, a non-caloric palatable fluid. For Experiment 2, adolescent males were tested during the age range corresponding to the transient yet stable increase in normalized SCM consumption in Experiment 1 (Fig 2). Adult male rats were tested at a later age to compare palatable reward intake in adolescents against a fully developed adult control group. Previous research has reported that adult males exhibit relatively stable normalized SCM intake during the currently employed age range [10]. Like Experiment 1, this experiment provided a strong characterization of age-related effects on palatable reward intake; however, in Experiment 2, the reward was non-caloric saccharin.

### Materials and methods

#### Animals and apparatus

Twenty-four experimentally naïve male Long Evans rats were used in this experiment: 12 adolescents and 12 adults. All rats were purchased from Harlan and arrived at the same time to ensure equivalent periods of acclimation. Rats in the adolescent group arrived with dams at PND 10, were weaned at PND 20, and began pre-training at PND 30. Adult rats arrived at PND 100–105 and began pre-training at PND 120–125 (Fig 1). As in Experiment 1, all rats were maintained with free access to home chow and water in the home cage and were handled for several days prior to behavioral testing. Animals in this experiment were euthanized via injections of sodium pentobarbital.

Behavioral testing occurred in the same chambers used in Experiment 1. However, rather than using a bottle for fluid delivery, saccharin solution was delivered via a syringe pump into a small (0.2 ml) acrylic fluid receptacle positioned within a recess in the front wall of the chamber. As in our previous studies [49, 50], a lickometer device was attached to the stainless steel input to the receptacle in order detect individual licking/lapping behaviors. A photobeam was positioned just above the recessed fluid receptacle to monitor approach behaviors.

#### Procedure

Rats underwent seven days of pre-training to familiarize them with saccharin and with drinking from the fluid receptacle. The first two days of pre-training involved 2 h of home-cage access to 0.20% saccharin from a bottle. This was followed by five days of 30 min access to 0.20% saccharin within the behavioral test chamber. Following pre-training, rats were given a series of tests in the chambers to characterize drinking behavior across a range of concentrations. Over three consecutive days, rats were given three 5-min saccharin consumption tests per day (nine tests total). Different saccharin concentrations (0.02, 0.20, or 1.00%) were used in each of the three daily tests. Test order (saccharin concentration) varied across both rats and days to minimize order effects. In all test sessions, an initial injection of saccharin solution (0.1 ml over 2 s) was delivered into the fluid receptacle at session onset. Subsequent saccharin injections (0.1 ml) were contingent on active fluid consumption. To avoid overfilling the fluid receptacle, no saccharin was delivered within 4 s of the last delivery. After this minimum inter-injection interval, saccharin was delivered as soon as the rat broke the photobeam above the receptacle and performed a licking response. No limit was placed on the amount of saccharin that rats could consume in these sessions.

#### Data analysis

Data analysis was generally as described in Experiment 1. Minor differences in approach are described below.

### Results and discussion

#### Training: Consumption rates

**Fig 5:**
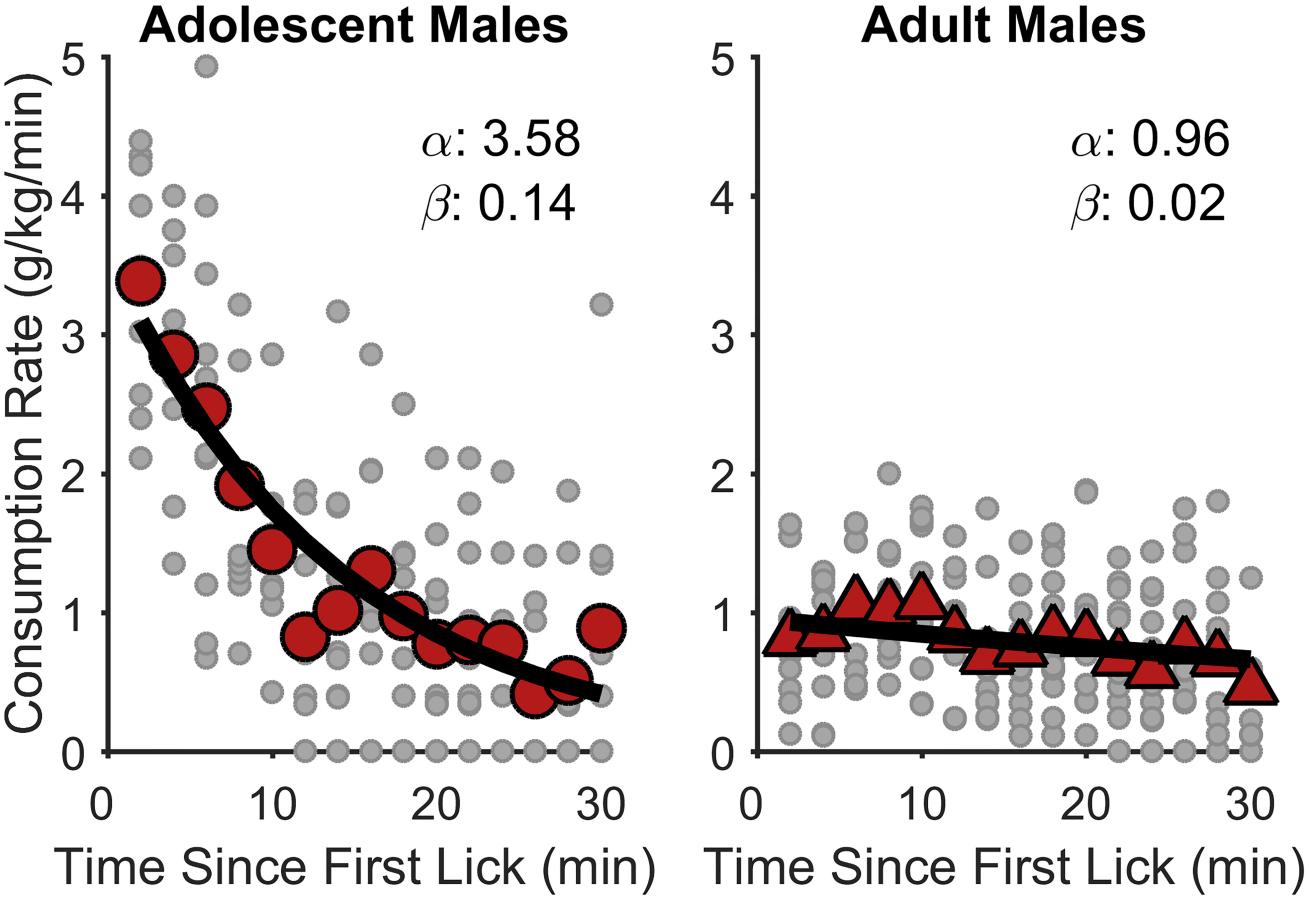
Normalized within-session saccharin consumption rates in Experiment 2. The large data points reflect group mean consumption rates in 2-min bins for adolescent males (left) and adult males (right). The small shaded data points represent individual rats’ data points from individual rats within that group. The thick line is the best-fitting negative exponential function. The within-panel α and β values are the fitted values of the corresponding exponential function (i.e., α is the y-intercept and a metric for reward palatability; β is the function’s decay and a metric for the induction of satiety). Adolescent males exhibited significantly greater initial consumption rates (α) and decay rates (β) than adult males, *p*s < .001.

Fig 5 shows the consumption rate of saccharin solution (normalized for body weight) as a function of time in the final session of pre-training. Because saccharin deliveries were only made when rats were actively consuming fluid from the receptacle, we used the amount of saccharin solution *delivered* as an estimate of *consumption*. Inspection of the fluid receptacles following individual test sessions confirmed that, by the last day of pre-training, all saccharin delivered into the fluid receptacle was consumed. Consumption rate was computed and plotted in 2-min bins. Both groups exhibited declining consumption rates across the session, though adolescent males (left) exhibited considerably larger initial consumption rates compared to adult males (right), in line with the results of Experiment 1 (Fig 3).

As in Experiment 1, nonlinear mixed-effects models ([Consumption Rate ~ α*exp(-(β*Time))] were fitted to these data to parse out the influence of palatability and satiety on normalized saccharin consumption. Analysis involved 330 observations. The fixed-effects structure for both α and β included the overall intercept and age group. The random-effects structure only included a by-subjects intercept for α. Full model output is shown in Table 2. Adolescent males exhibited significantly greater initial consumption rates, *t*(305) = 7.91, *p* < .001, and consumption decay rates, *t*(305) = 7.75, *p* < .001, compared to adult males (Fig 5). In accordance with Experiment 1, these data provide further evidence that (1) the hedonic value attributed to palatable fluids is heightened in adolescent male rats and (2) that this effect is not dependent on the use of caloric stimuli.

#### Testing: Concentration curve

**Fig 6:**
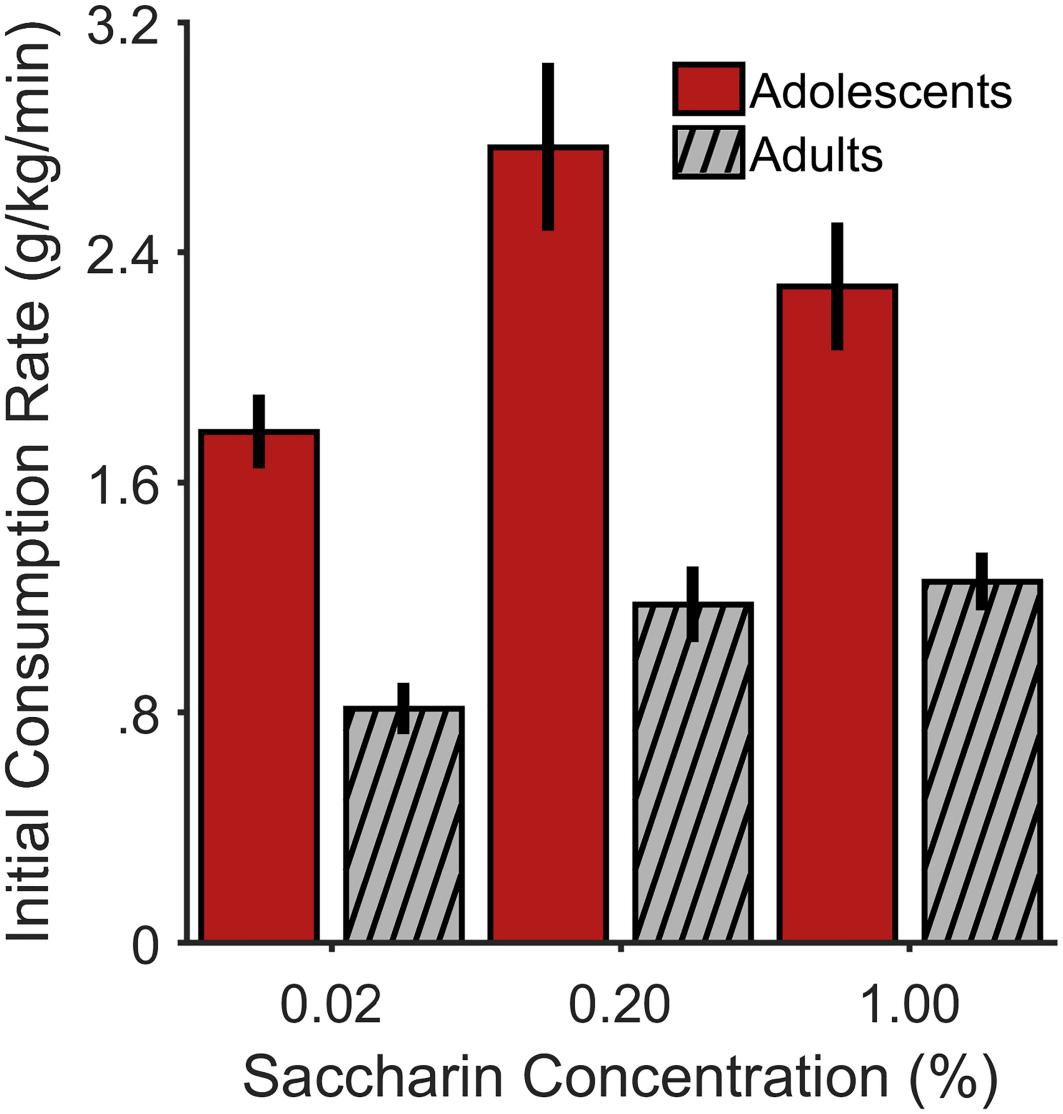
Initial normalized saccharin consumption rates as a function of saccharin concentration for each age group in Experiment 2. The bars represent group means and the error bars represent +/- 1 between-subjects SEM relative to the group mean.

Fig 6 shows mean normalized saccharin consumption rates for adolescent and adult males in the initial 2 min of intake for test sessions in which saccharin concentration was varied. These data were collapsed across tests of the same saccharin concentration. To determine the impact of palatable reward concentration on orosensory reward processing, we focused this analysis on the first 2 min of consumption from each test, as drinking during this period is predominantly driven by orosensory reward and minimizes the influence of satiety [51–56]. These data were not obtained through model fitting of the negative exponential function. However, the mean initial consumption rates plotted in Fig 6 are analogous to the fitted α values of the exponential function. As seen in Fig 6, adolescent males exhibited considerably higher rates of normalized consumption compared to adult males for each saccharin concentration.

Linear mixed effects model analysis involved 196 observations, and included a fixed-effects structure of the intercept, age group, and concentration. The random-effects structure only included a by-subjects intercept. To correct for positive skewness of the data, initial normalized consumption rates were square-root transformed. The plotted data are in linear space for ease of interpretation (Fig 6). Full model output is shown in Table 3. Notably, the adolescent males exhibited significantly greater initial saccharin consumption rates compared to adult males, *t*(192) = 5.78, *p* < .001. The best-fitting model did not include an age group by concentration interaction. Specifically, the addition of this interaction to the reported model increased the AIC by 7.93, which provided insufficient evidence for its possible inclusion [27]. Therefore, this interaction did not contribute to the fit of the model; in other words, the effect of age group was not moderated by saccharin concentration. Accordingly, male adolescent rats showed a pronounced elevation in palatability-driven feeding across a broad range of saccharin concentrations. Indeed, as in Experiment 1, the patterns of normalized chow and water intake could not reasonably account for these data (S2 Fig).

**Table 3:** Linear mixed-effects model output for the analysis of normalized saccharin consumption rates in concentration curve testing in Experiment 2.

	*t*(192)	*P*	*b*	95% CI
Intercept	30.95	< .001	1.24	[1.16, 1.31]
Age Group (Adult/Adolescent)	5.78	< .001	0.23	[0.15, 0.31]
Concentration (0.02)	-5.47	< .001	-0.16	[-0.21, -0.10]
Concentration (0.20)	3.67	< .001	0.10	[0.05, 0.16]

Note: The categorical variable of age group was effect coded with Adult/Adolescent (Age Group) as -1/+1. Concentration was also a categorical variable, and 1.00% served as the reference level.

## General discussion

Individual differences in palatable reward consumption has become a particularly important area of study, given the prevalence of obesity in the United States [57] and a rising prevalence among children [58]. Both drug addiction and obesity have been suggested to involve similar neurobiological substrates, and may be driven by stronger preferences for or sensitivities to reward [6, 59]. Accordingly, greater reward sensitivity in adolescents may facilitate engagement in maladaptive behaviors. Also, differential exposure to sucrose during adolescence may have strong impacts on behavior in adulthood [60, 61]. As described above, adolescent risk taking may be driven by adolescent hypersensitivity to and overvaluation of appetitive stimuli [6]. Accordingly, greater understanding of adolescent reward processing (e.g., reward palatability) relative to that of adults has potential implications for future age- and sex-dependent treatments to alleviate any elevated propensities for risk-taking behaviors [62].

Accordingly, this study investigated differences in voluntary palatable reward consumption between adolescent and adult rats. Here, male, but not female, adolescent rats exhibited elevated consumption of sweet fluid, a transient effect that was limited to PND 40–50, which corresponds to early to mid-puberty in adolescent male rats (also see [10]). Importantly, this effect was not reasonably explained by comparable differences in raw palatable reward intake (S1 Fig), as adult rats consumed more palatable reward than adolescent rats without respect to body weight. Also, while adolescent rats exhibited greater normalized home chow and water intake compared to adult rats, the longitudinal changes in these data did not mirror those of normalized palatable reward intake (S2 Fig), suggesting a unique effect of palatability on reward consumption in adolescent rats, specifically males. Accordingly, because various factors may govern the total amount of food that an animal will freely consume, we applied a well-established analytical approach to determine rats’ initial rate of palatable fluid intake, which is a more selective measure of the influence of orosensory reward (palatability) on feeding [14, 16]. Adolescent male rats exhibited an initial rate of feeding that, in some cases, was over 3.5 times that of adult male rats when controlling for body weight (Fig 5), suggesting greater hedonic value of reward in adolescent males [6, 10, 11]. Interestingly, faster rates of eating have been linked to fatty liver disease [63], obesity [64], and self-reports of reduced satiety despite greater energy intake [65, 66], suggesting that greater understanding of the temporal dynamics of adolescent feeding behavior has critical health implications (see [58]).

Importantly, heightened palatability-driven feeding in adolescent males was observed under free-feeding (non-deprived) conditions and was apparent regardless of whether the fluid being consumed was caloric (SCM; Experiment 1) or not (saccharin; Experiment 2), suggesting that ontogenetic differences in metabolism or nutritional demands were not major contributors to this effect. Relatedly, this result was unlikely due to an underlying insensitivity to the feeding-suppressive effects of post-ingestive satiety: male adolescent rats showed a more rapid onset of satiety (i.e., larger β decay rate) than the other groups, likely driven by their vigorous feeding early in test sessions. This relationship between initial feeding rate and rate of satiety has been described previously [46] and is further supported by our finding that these measures were highly correlated with each other (Fig 4). Although male adolescent rats differed from other groups in both feeding parameters, we found that these measures were differentially associated with overall intake (Fig 4). Whereas the rate of initial feeding was strongly associated with total SCM consumption, no such relationship was found between the rate of satiety and total consumption. Thus, the elevation in hedonic value during stages of adolescence in male rats is more likely governed by changes in orosensory stimulation.

Caloric and non-caloric sweetened solutions may produce differences in feeding behavior [67], therefore warranting investigation of palatable reward consumption of both types of solutions. Here, adolescent males exhibited greater initial rates of consumption compared to adult males, even at higher concentrations of saccharin (Fig 6). As the preference-aversion function of saccharin indicates that it becomes more aversive at larger concentrations (e.g., [37, 67]), these results suggest that the adolescent males may be (1) more sensitive to the appetitive components of saccharin, and/or (2) less sensitive to the aversive components of saccharin [4, 6, 11]. Interestingly, adolescent males have been shown to exhibit faster acquisition of conditioned responding in partial-reinforcement schedules compared to adult males [68], suggesting that adolescents may be more attentive to the appetitive aspects of a reinforcement schedule (i.e., reinforcer delivery) as opposed to its aversive aspects (i.e., reinforcer omission) (also see [9]). Such weighting of appetitive over aversive factors may thus explain the prevalence of gambling in adolescents [69]. Accordingly, elevated hedonic (appetitive) value of reward in adolescents may have implications for risky and impulsive behavioral tendencies in adolescents [8, 69–71].

Collectively, these findings support the hypothesis that reward stimuli are attributed greater value during adolescence [3, 4, 6], potentially contributing to the heightened levels of risky reward-seeking behaviors associated with this stage of development [1–4]. A tendency to assign greater value to reward stimuli may partially contribute to these exaggerated levels of risky reward-seeking behaviors [4, 6]. Greater sensitivity to orosensory reward in adolescent male rats compared to adolescent female rats is interesting, particularly given reports of sex differences in human adolescent risk taking, in which boys tend to be more willing to accept risk than girls [72, 73]. On the other hand, in Experiment 1, adult females exhibited greater initial consumption rates (α; i.e., reward palatability) than adult males, which, within the current framework, would suggest greater risk-taking in adult females versus males (Fig 3). Interestingly, while the “gender gap” in risk-taking propensities may decrease with age [12], more recent research in humans has suggested that young-adult women may be more likely than young-adult men to exhibit a low-cost risky behavior that has mostly positive and very minimal negative consequences [74]. Accordingly, if initial licking behavior in the present experiment can be viewed as a low-cost behavior with primarily positive results, then perhaps there are noteworthy yet understudied reversals in risk-taking propensities from adolescence to adulthood that depend on sex and the type of risk-taking behavior under evaluation. Alternatively, elevated orosensory reward processing yet comparable risk-taking levels in adult females versus adult males may suggest that this mechanism does not solely account for sex differences in risk-taking. Ultimately, future research will elucidate these important questions, as greater tolerance for risk may have important health implications. Whether this gender gap in adolescent risk taking is related to a fundamental sex difference in reward processing requires further research, as other factors are also likely to contribute to sex differences in adolescent risk taking (e.g., risk evaluation, changing gender roles, peer pressure) [12].

While elevated palatable food intake displayed by adolescent male rats found here and in previous research [10] is likely related to a difference in orosensory reward processing and not an insensitivity to satiety, other psychological processes are also believed to contribute to feeding behavior (e.g., incentive motivation, habit formation) [34, 75]. While definitive methods for measuring the individual contributions of these processes to feeding have yet to be established, there is some indication that the impact of these *learned* components of feeding exert an influence that is discriminable from the more direct influence of food palatability. For example, while food palatability has been shown to influence how long animals spend actively feeding when they are in direct contact with food (e.g., [56]), it is believed that food-associated environmental cues exert a distinct motivational influence, triggering new bouts of feeding when animals are not directly engaged in such behavior. Indeed, cues associated with palatable food appear to be particularly effective promoting feeding in fully satiated animals [76–79]. Regardless of whether such cues exert their influence on feeding through incentive motivation (e.g., by inducing a “craving”) or by directly eliciting a feeding habit, one would expect this effect to be primarily apparent later in the session, when the suppressive influence of satiety is greatest. Thus, a greater initial palatable fluid intake and a more rapid and sustained suppression of feeding in response to satiety in adolescent males relative to adults suggests that these groups differed in hedonic emotional responses during palatable food consumption rather than in their level of cue-motivated or habitual feeding. However, further research using more precise behavioral methods is warranted to determine the degree to which these latter components of feeding are impacted by adolescent development.

Our findings suggest that the neurobiological systems underlying reward processing undergo pronounced, sex-specific developmental adaptations during adolescence. During adolescence, there is considerable overproduction and pruning of striatal and prefrontal dopamine (DA) receptors [80–82], which appears to be more prominent in males than in females and overlaps with male puberty [83, 84], with receptor levels peaking around PND 40. Although DA signaling plays a crucial role in various aspects of decision making and motivated behavior (e.g., [85]) and dopamine activity peaks in adolescence (see [86]), studies using selective behavioral measures of food palatability suggest that DA does not play a crucial role in this aspect of reward processing [34]. However, there are considerable age-dependent changes in endogenous opioid peptide [87–90] and cannabinoid systems [91, 92], and these systems have been more directly implicated in palatability-driven feeding and related measures of reward processing [49, 93–98]. Interestingly, adult females may exhibit upregulated endocannabinoid function [91], which is congruent with their greater normalized SCM consumption compared to adult males shown here and previous research that suggested stronger preference for sweet solutions in adult female versus adult male rats [13, 99].

In conclusion, the present results demonstrate that adolescent males attribute greater hedonic value to sweet rewards compared to the other age/sex groups (adolescent females, adult males and females). These results corroborate previous research (e.g., [6]), but also expand our current understanding of adolescent reward sensitivity and the hedonic influences of palatable reward consumption in adolescence. As the present experiments compared groups as a function of PND, we believe that future research considering sex differences in puberty onset will also provide valuable insight into developmental changes in reward processing. Here, the developmental phase of interest primarily corresponded to early to mid-puberty in adolescent males. Given earlier puberty onset in females than males (see [32]), additional testing in female rats at earlier PNDs will strengthen our understanding of the sex specificity of the current results. Moreover, whether adolescent males’ transient uptick in normalized SCM consumption is time-locked to age and/or puberty-induced hormonal changes would advance our knowledge of the developmental trajectory of reward processing. Continued research of palatable reward intake in adolescent and adult male and female rats may ultimately unveil the critical mechanisms driving maladaptive decision making in adolescents (e.g., [2, 100]).

## Supporting information

S1 Fig SCM consumption and body weights in Experiment 1. Top: Group mean raw consumption data (g) (+/- 1 between-subjects SEM). Bottom: Group mean body weights (g) (+/- 1 between-subjects SEM). In both panels, the abscissa is session.
(TIF)

S2 Fig Normalized home-cage chow and water consumption in Experiments 1 and 2. Left: Group means (+/- 1 between-subjects SEM) of home-cage chow normalized consumption data (g/kg) over a 24-hr access period measured weekly over the course of testing in Experiment 1. Right: Group means (+/- 1 between-subjects SEM) of home-cage normalized chow and water consumption data (g/kg) over a 24-hr access period measured weekly over the course of testing in Experiment 2. In both panels, the abscissa refers to individual measurements, but is labeled to refer to the groups’ ages at each measurement (adolescent PNDs are outside of the brackets; adult PNDS are inside the brackets). In Experiment 1, there were two more measurements of chow intake in adolescents versus adults.
(TIF)
