# Supplementary figures and images for: Sex-specific enhancement of palatability-driven feeding in adolescent rats
Source: PLoS One. 2017 Jul 14;12(7):e0180907. doi: 10.1371/journal.pone.0180907 (PMC5510835; doi:10.1371/journal.pone.0180907)

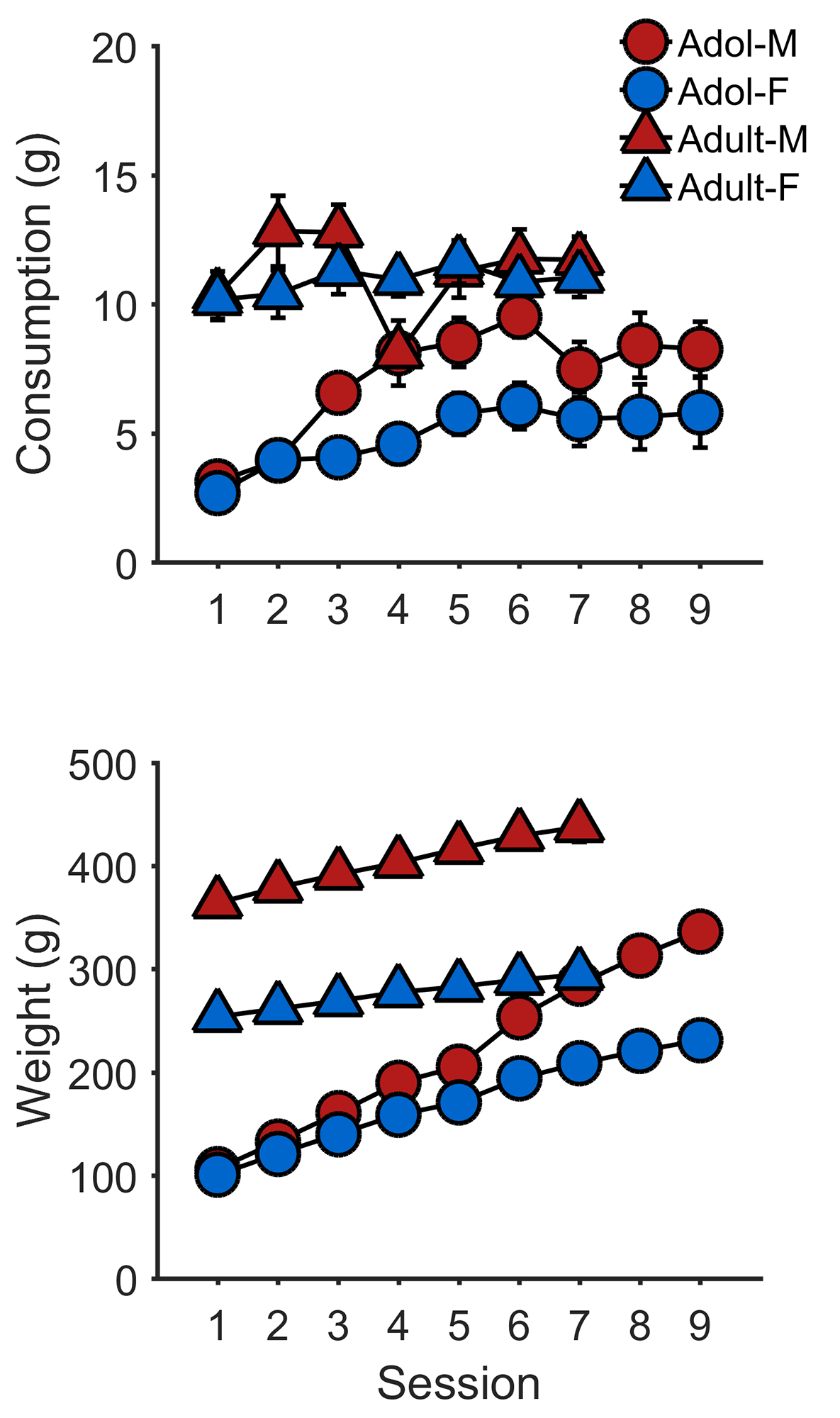

Supplement: S1 Fig — Top: Group mean raw consumption data (g) (+/- 1 between-subjects SEM). Bottom: Group mean body weights (g) (+/- 1 between-subjects SEM). In both panels, the abscissa is session. (TIF) [file pone.0180907.s001.tif]

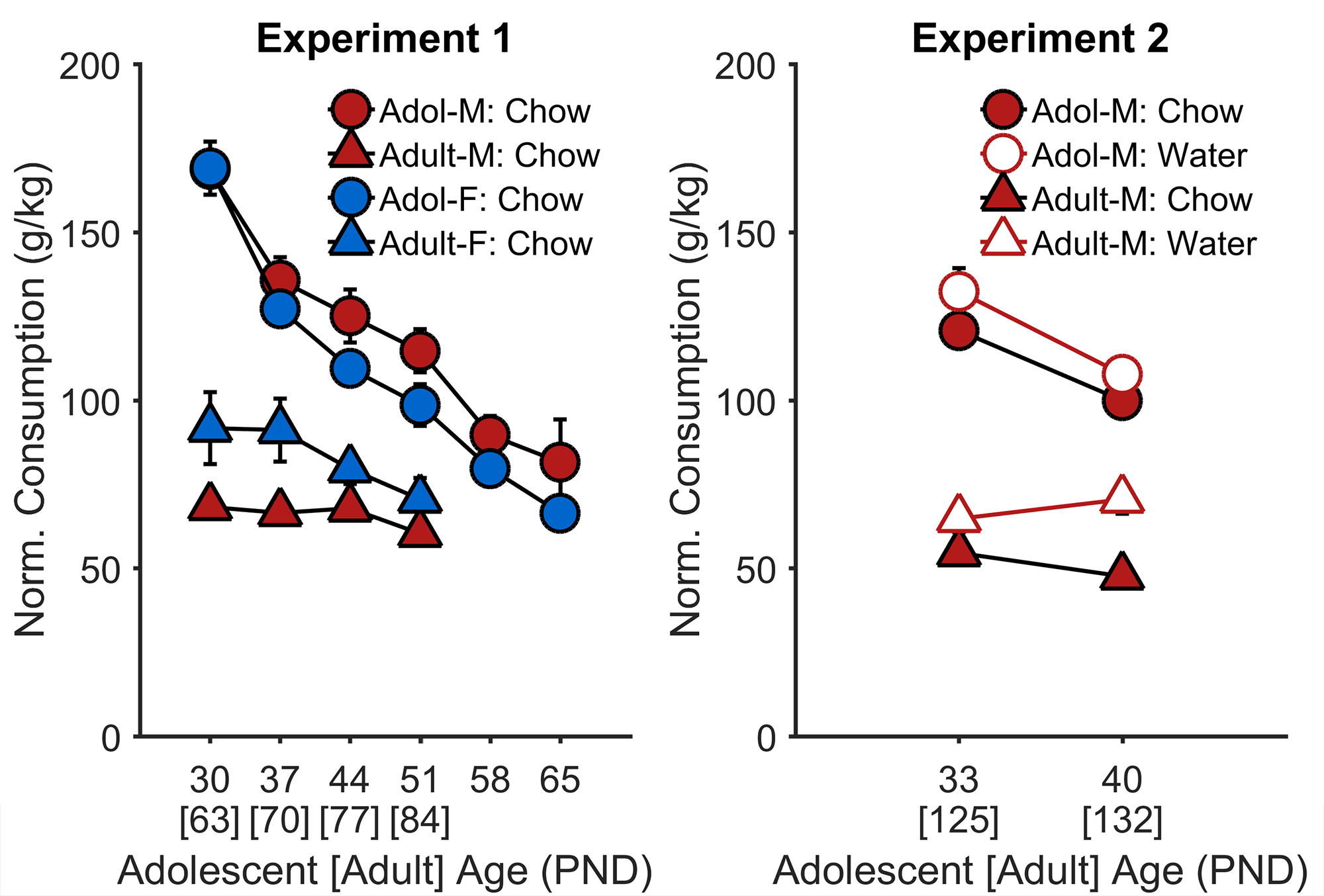

Supplement: S2 Fig — Left: Group means (+/- 1 between-subjects SEM) of home-cage chow normalized consumption data (g/kg) over a 24-hr access period measured weekly over the course of testing in Experiment 1. Right: Group means (+/- 1 between-subjects SEM) of home-cage normalized chow and water consumption data (g/kg) over a 24-hr access period measured weekly over the course of testing in Experiment 2. In both panels, the abscissa refers to individual measurements, but is labeled to refer to the groups’ ages at each measurement (adolescent PNDs are outside of the brackets; adult PNDS are inside the brackets). In Experiment 1, there were two more measurements of chow intake in adolescents versus adults. (TIF) [file pone.0180907.s002.tif]
